# Lack of Associations Between Dietary Intake and Gastrointestinal Symptoms in Autism Spectrum Disorder

**DOI:** 10.3389/fpsyt.2019.00528

**Published:** 2019-07-25

**Authors:** Bradley J. Ferguson, Kristen Dovgan, Danielle Severns, Shannon Martin, Sarah Marler, Kara Gross Margolis, Margaret L. Bauman, Jeremy Veenstra-VanderWeele, Kristin Sohl, David Q. Beversdorf

**Affiliations:** ^1^Department of Health Psychology, School of Health Professions, University of Missouri, Columbia, MO, United States; ^2^Thompson Center for Autism & Neurodevelopmental Disorders, Columbia, MO, United States; ^3^Department of Radiology, University of Missouri School of Medicine, Columbia, MO, United States; ^4^Department of Psychology, Marist College, Poughkeepsie, NY, United States; ^5^School of Medicine, University of Missouri, Columbia, MO, United States; ^6^Department of Psychological Sciences, University of Missouri, Columbia, MO, United States; ^7^Medical Exploration of Neurodevelopmental Disorders Clinic (MEND), Vanderbilt University Medical Center, Nashville, TN, United States; ^8^Department of Pediatrics, Division of Pediatric Gastroenterology, Hepatology, & Nutrition & Morgan Stanley Children’s Hospital, Columbia University Medical Center, Columbia, MO, United States; ^9^Department of Anatomy and Neurobiology, Boston University School of Medicine, Boston, MA, United States; ^10^Department of Psychiatry, Columbia University, Columbia, MO, United States; ^11^Department of Child Health, University of Missouri, Columbia, MO, United States; ^12^Departments of Neurology & Psychological Sciences, William and Nancy Thompson Endowed Chair in Radiology, University of Missouri, Columbia, MO, United States

**Keywords:** autism spectrum disorder (ASD), gastrointestinal symptoms, dietary intake, omega-3 fatty acids, micro- and macronutrients

## Abstract

**Background:** Many individuals with autism spectrum disorder (ASD) have significant gastrointestinal (GI) symptoms, but their etiology is currently unknown. Dietary interventions are common in children and adolescents with ASD, including diets with increased omega-3 fatty acids or diets free of gluten and/or casein, which may also impact GI symptoms and nutrition. However, little is known about the relationship between nutritional intake and GI symptomatology in ASD. The objective of this study was to assess the relationships between GI symptoms, omega-3 intake, micronutrients, and macronutrients in children with ASD.

**Methods:** A total of 120 children diagnosed with ASD participated in this multisite study. A food frequency questionnaire was completed by the patient’s caretaker. The USDA Food Composition Database was utilized to provide nutritional data for the food items consumed by each participant. GI symptomatology was assessed using a validated questionnaire on pediatric gastrointestinal symptoms.

**Results:** There were no significant associations between GI symptoms and the amount of omega-3 fatty acids and/or other micro- and macronutrients contained in the diet.

**Conclusions:** This study suggests that dietary variations do not appear to drive GI symptoms, nor do GI symptoms drive dietary variations in those with ASD, although causation cannot be determined with this observational assessment. Furthermore, there may be other factors associated with lower GI tract symptoms in ASD, such as increased stress response.

## Introduction

Autism spectrum disorder (ASD) is a neurodevelopmental condition characterized by persistent deficits in social communication and social interaction, as well as restricted and repetitive patterns of behavior that present during early development and result in clinically significant impairment (1). Research has shown that children with ASD tend to have more gastrointestinal (GI) symptoms than their typically developing peers (2–6), especially for constipation, diarrhea, and abdominal pain (2, 7–9). A review of the literature in 2010 indicated that the proportion of ASD individuals having co-occurring GI problems may range from 9 to 91% (10), the reported variations in prevalence rates potentially resulting from differences in diagnostic methods used to assess GI symptoms in this population. Despite the relatively high rates of GI symptoms in ASD, the etiology is poorly understood. Therefore, it is important to explore the role of dietary associations with GI symptoms in ASD given the potential for certain diets to be associated with GI dysfunction in ASD.

Many individuals with ASD have used complementary and alternative medicine (CAM) approaches, including dietary changes, as part of their treatment for the core ASD symptoms, as well as GI disturbance, sleep problems, or in the promotion of general health (11). As such, changes in diet may affect GI functioning in ASD. Some studies have shown that children with ASD may be deficient in micro- and macronutrients (12–15), as well as iron (16), which could result from altered GI function and/or potentially impact GI symptoms. Furthermore, many parents and caretakers have employed the use of gluten- and casein-free (GFCF) diets (17) that seem to have mixed effects on core ASD symptoms (18) and GI symptoms (19–23) in ASD. In addition, many families also administer omega-3 fatty acids in the hope of deriving benefit, but the results from randomized, placebo-controlled clinical trials of omega-3 supplementation in ASD are also mixed in most cases (24, 25). In addition, limited dietary intake and selective food preferences, common among individuals with ASD (26), can result in nutritional deficiencies or other problems that could potentially interact with GI symptoms, either contributing to these symptoms or emerging in response to them.

As such, a better understanding of the association between dietary intake on GI functioning in ASD is of interest, especially given the implications for treatment. The focus of the present study is to assess the associations between approximate omega-3 intake and micro- and macronutrient intake over the prior month and self- and parent-reported GI symptoms in individuals with ASD with the goal of determining whether dietary factors may be related to GI symptomatology.

## Methods

A total of 120 patients with ASD (mean age = 11.8, *SD* = 3.8, range = 6–18, 108 male, 92.5% Caucasian, mean full-scale intelligence quotient = 84, *SD* = 22.6, range = 36–130) participated in this study. Patients were recruited sequentially from individuals enrolled in the Autism Speaks Autism Treatment Network (AS-ATN) registries at the University of Missouri Thompson Center for Autism & Neurodevelopmental Disorders in Columbia, Missouri, and at the Vanderbilt Kennedy Center and Monroe Carrell Jr. Children’s Hospital at Vanderbilt University in Nashville, Tennessee. To expand the sample, additional patients who were not enrolled in the AS-ATN were recruited from clinic patients at each site. Diagnosis of ASD was made based on *Diagnostic and Statistical Manual for Mental Disorders* *IV-TR* criteria (27) and the administration of the Autism Diagnostic Observation Schedule (ADOS) (28). Patients with known genetic or metabolic disorders or bleeding disorders were excluded from this study, as an associated portion of this project involved drawing blood. A more detailed explanation of the inclusionary and exclusionary criteria can be found elsewhere (29, 30). This study was carried out in accordance with the recommendations of the Institutional Review Boards at the University of Missouri and Vanderbilt University, with written informed consent from all participants over the age of 18 and consent from the parent/guardian and assent from those under the age of 18. All participants gave written informed consent in accordance with the Declaration of Helsinki. The study protocol was approved by the Institutional Review Boards at the University of Missouri and Vanderbilt University.

### Assessment of Gastrointestinal Symptoms

Gastrointestinal symptoms were assessed based on parent or self-report using the Questionnaire of Pediatric Gastrointestinal Symptomatology-Rome III (QPGS-RIII) (31). Patients who were over the age of 18 and able to provide an accurate account of their GI symptoms, as determined by asking the caretaker and/or the patient if they could reliably report their GI symptoms over the month before participation in the study, completed the self-report version of the QPGS-RIII. Otherwise, the QPGS-RIII was completed by a parent or caretaker that could provide a reliable account of the patient’s GI functioning over the month before their participation in the study. A scoring rubric previously created by the research team was used to create continuous variables for upper and lower GI tract symptoms over the past month (29). Briefly, items from the QPGS-RIII were sorted into upper and lower GI tract symptoms, and the scores for each were summed to reflect an overall upper and lower GI score for each patient. Greater QPGS-RIII scores indicate greater frequency, severity, and duration of GI symptoms.

### Assessment of Nutritional Intake

Omega-3 nutritional intake during this same period of time (1 month before entering the study) for each patient was assessed using a food frequency questionnaire (FFQ) that was designed to assess omega-3 fatty acid intake as well as information that could be utilized to calculate micro- and macronutrient intake (32). The 152-item FFQ was developed based on foods that contain 10 mg of n-3 fatty acids/medium serving from fish, animal, and plant sources. The FFQ was either completed by the patient’s parent or caregiver or by the patient. Responses were analyzed for nutritional intake using the online, publicly available United States Department of Agriculture Food Composition Database (33), which provides nutrient information for specific foods. A total monthly estimate of the patient’s nutritional intake was calculated by summing the nutrient information for each food based on the serving size and frequency of consumption. Total nutrient scores were created for both University of Missouri and Vanderbilt AS-ATN sites to determine if differences exist between midwestern and southern region diets. The individuals scoring the FFQs were blinded to the patient’s GI status.

## Results

### Gastrointestinal Symptoms

Both sites had similar number of patients with upper and lower GI tract problems [*t*(113) = 1.608, *p* = 0.111]. Therefore, the two populations were pooled for the primary comparisons. In addition, upper GI tract problems were significantly correlated with lower GI tract problems (*r* = 0.411, *p* < 0.001). The most common GI disorder reported by the participants was functional constipation (42.5%), followed by irritable bowel syndrome (11.7%) and lower bowel pain associated with bowel symptoms (9.2%). A more detailed description of the GI disorders experienced by this study population as well as how the GI scores were calculated can be found in previous reports (29, 30).

### Omega-3 and Dietary Nutrient Intake

See **Table 1** for approximate mean monthly nutrient intake values across both the University of Missouri and Vanderbilt sites. First, as the nutrient variables were significantly skewed and non-normal, nonparametric Spearman rank correlations were conducted on the full dataset (i.e., without removing outliers), which do not make assumptions about the normal distribution of the data. In this way, we could assess the potential contribution of any extreme diets and picky eaters. Total GI tract symptoms were not significantly correlated with fatty acids (*r* *_s_* = 0.145, *p* = 0.20), gluten (*r* *_s_* = 0.114, *p* = 0.336), casein (*r* *_s_* = −0.104, *p* = 0.357), water (*r* *_s_* = −0.059, *p* = 0.605), calories (*r* *_s_* = 0.137, *p* = 0.225), protein (*r* *_s_* = 0.113, *p* = 0.319), fats (*r* *_s_* = 0.147, *p* = 0.193), carbohydrates (*r* *_s_* = 0.088, *p* = 0.438), sugar (*r* *_s_* = 0.093, *p* = 0.412), vitamins (*r* *_s_* = 0.005, *p* = 0.962), minerals (*r* *_s_* = 0.068, *p* = 0.550), or cholesterol (*r* *_s_* = 0.132, *p* = 0.244). However, fiber was positively correlated with upper and lower GI tract symptoms (*r* *_s_* = 0.243, *p* = 0.030) when outliers were included. Furthermore, it is not unusual for extreme points to increase the strength of a correlation, and the sample included three participants who consumed over 656 g of fiber in the past month.

**Table 1:** Mean monthly nutrient intake values for the full sample.

	Mean	SD	Range (with outliers)^a^	N
Rome III Upper GI Score	4.5	4.6	0–20	116
Rome III Lower GI Score	17.6	11.8	0–50	118
Casein (g)	39.0	35.2	0–247	116
Gluten (g)	21.6	19.7	0–96	110
Water (g)	15,157.3	8,678.9	1,395.36–79,019.23	116
Energy (kcal)	19,410.6	8,187.4	6,700–72,793	116
Protein (g)	1,082.9	439.4	395.42–3,929.90	114
Total lipid fat (g)	766.6	352	169.59–2,897.07	115
Carbohydrate (by difference; g)	2,021.5	970.5	296.92–8,394.04	114
Dietary fiber (total; g)	234	147.3	2.20–865.95	117
Sugars (total; g)	779.3	538.3	25.14–4,447.32	114
Calcium (mg)	16,710	11,842	1,067–107,330	116
Iron (mg)	113.3	64.4	26.65–386.99	109
Magnesium (mg)	3,805.8	1,990.4	818–17,700	117
Phosphorous (mg)	20,161	10,452.2	4,614–91,666.50	117
Potassium (mg)	40,703.2	20,063.2	7,936–196,649	117
Sodium (mg)	24,292.4	10,906.4	6,224–90,283	113
Zinc (mg)	143.4	66.8	28.03–544.18	115
Vitamin C (total ascorbic acid; mg)	1,178.6	1,067.8	2.80–9,534.95	113
Thiamin (mg)	16.5	7.5	3.73–61.41	115
Riboflavin (mg)	26.3	15.8	3.54–137.25	117
Niacin (mg)	250.2	107.0	72.74–714.32	115
Vitamin B6 (mg)	28.7	15.5	6.84–189.69	112
Folate (DFE; µg)	3,436.4	2,094.2	384–23,399	114
Vitamin B12 (µg)	68.6	39.2	1.26–305.51	113
Vitamin A (RAE; µg)	8,091.1	5,379.1	126–46,020	115
Vitamin A (IU)	57,991.1	44,091.6	726–470,724.50	112
Vitamin E (alpha tocopherol; mg)	98.3	52.6	7.83–486.53	115
Vitamin D2 + D3 (µg)	75.7	67.6	2.50–339	114
Vitamin D (IU)	2,984.2	2,694.5	82.50–13,212.56	114
Vitamin K (phylloquinone; µg)	981.3	714.1	37–5,649.75	115
Total saturated fatty acids (g)	258.9	130.7	34.59–1,391.45	115
Total monounsaturated fatty acids (g)	263.7	128.6	43.68–1,173.70	112
Total polyunsaturated fatty acids (g)	161.4	79.1	16.68–537.98	115
Total trans fatty acids (g)	6.5	4.9	0.54–84.90	110
Cholesterol (mg)	3,139.3	1,535.5	50–10,945	111

a Note that the range reflects the full sample, including outliers.

Next, we wished to reanalyze the data excluding the outliers. As such, 173 outlier values (3.7% of the data) were removed using the interquartile range rule (i.e., values >1.5 times the interquartile range), creating a normally distributed dataset that could be analyzed using Pearson correlations. See **Table 1** for the number of patients remaining for each micro- and macronutrient. Estimated omega-3 fatty acids were not significantly correlated with upper or lower GI tract symptoms across both sites (*r* = 0.10, *p* = 0.304). Furthermore, upper and lower GI tract symptoms were not significantly correlated with the consumption of gluten, casein, water, calories, protein, fats, carbohydrates, fiber, sugar, or any vitamins, minerals, or cholesterol. See **Table 2** for Pearson correlations between upper and lower GI tract problems and each nutrient intake value.

**Table 2:** Correlation matrix for nutrient intake values and Questionnaire of Pediatric Gastrointestinal Symptomatology-Rome III (QPGS-RIII) upper and lower gastrointestinal (GI) tract symptom scores.

	Rome III Upper GI Score	Rome III Lower GI Score
Rome III Upper GI Score	1	0.411**
Rome III Lower GI Score	0.411**	1
Casein (g)	−0.182	−0.084
Gluten (g)	0.003	0.122
Water (g)	−0.034	−0.032
Energy (kcal)	0.071	0.107
Protein (g)	−0.004	0.131
Total lipid fat (g)	0.095	0.142
Carbohydrate (by difference; g)	0.067	0.121
Dietary fiber (total; g)	0.180	0.166
Sugars (total; g)	0.088	0.096
Calcium (mg)	−0.148	−0.020
Iron (mg)	0.155	0.150
Magnesium (mg)	0.021	0.091
Phosphorous (mg)	−0.036	0.026
Potassium (mg)	−0.022	0.041
Sodium (mg)	0.017	0.134
Zinc (mg)	0.093	0.176
Vitamin C (total ascorbic acid; mg)	0.176	0.093
Thiamin (mg)	0.008	0.055
Riboflavin (mg)	−0.087	−0.015
Niacin (mg)	0.037	0.081
Vitamin B6 (mg)	−0.017	−0.006
Folate (DFE; µg)	0.096	0.126
Vitamin B12 (µg)	−0.016	0.012
Vitamin A (RAE; µg)	−0.068	−0.002
Vitamin A (IU)	−0.011	0.084
Vitamin E (alpha tocopherol; mg)	0.096	0.153
Vitamin D2 + D3 (µg)	−0.086	−0.040
Vitamin D (IU)	−0.112	−0.046
Vitamin K (phylloquinone; µg)	0.127	0.107
Total saturated fatty acids (g)	−0.009	0.039
Total monounsaturated fatty acids (g)	0.119	0.137
Total polyunsaturated fatty acids (g)	0.140	0.112
Total trans fatty acids (g)	0.111	0.123
Cholesterol (mg)	0.033	0.076

**p < 0.001.

## Discussion

Previous research from this multidisciplinary, investigative team found associations between the stress response and GI symptoms among those with ASD (29, 30). General nutritional intake as well as consuming foods high in n3-PUFA may also, however, affect GI symptoms, or GI symptoms might affect diet. Thus, we sought to examine the association between nutritional intake and GI symptoms in the same group of individuals from the aforementioned study. The present study was conducted to specifically examine the effects of diets that are high in n-3 PUFA on GI problems in those with ASD. The results from this multisite study indicate no association between consumption of a diet that is high in n-3 PUFA and upper or lower GI tract symptoms in the study sample. Furthermore, micro- and macronutrients contained in the diet were also not significantly associated with upper and/or lower GI tract symptoms in the sample. These results suggest that previous relationships between stress reactivity and GI symptomatology are not due to dietary factors, at least those assessed herein, and begins to provide evidence against the concept of dietary factors impacting GI symptomatology or of GI symptomatology impacting diet in children with ASD. The isolated finding of a positive correlation between fiber intake and GI symptomatology before excluding outliers may result from unsuccessful attempts to manage the GI symptoms with high fiber intake. Indeed, dietary fiber has been shown to be associated with GI symptoms of abdominal pain, bloating, and constipation, flatulence, and diarrhea (34, 35). Therefore, parents, caratakers, and clinicians should be aware of this finding as well as recommended fiber intake (36, 37) when considering treatment of abdominal pain and constipation in children with ASD.

There are a number of limitations in this study that should be addressed. First, the study did not examine the effects of altered diets on GI functioning in the sample. As many individuals with ASD have altered diets, it is a possibile that a subgroup of autistic individuals with altered diets may have concomitant alterations in GI functioning. Thus, future research should examine the effects of altred diets on GI symptoms in ASD. Second, the present study utilized a food frequency questionnaire that contained a limited number of food items. While the questionnaire contains a wide range of food items, it is not exhaustive. Furthermore, it is not clear if taking dietary supplements by participants could be related to their GI symptoms. Future research may wish to utilize a food diary to log food items and amounts consumed per day as well as assess whether or not the participant is taking dietary supplements in an attempt to reduce ASD symptoms. Third, the sample was largely male and Caucasian, and so it is not clear if the results transfer to female and other ethnicities. Fourth, the QPGS depends on an informant, usually a parent, to answer a questionnaire regarding their child’s GI functioning, including identification of the location of abdominal discomfort. Given that many children with ASD are nonverbal or have limited verbal abilities, it is possible that the GI scores may not be accurate for all participants. Future GI investigations should utilize formal gastroenterological evaluations or, at minimum, consider the use of ASD-specific measures of GI symptoms (38). Finally, the results presented herein will need to be replicated before drawing conclusions regarding the relationship between diet and GI disorders in ASD in the broader population. Larger samples would also allow incorporation of other co-occurring conditions to examine their relationships with the results from this study, as well as a better ability to recognize subtypes in the heterogenous ASD population.

## Conclusion

The results from this study indicate no significant associations between dietary omega-3 and GI symptoms as well as dietary micro- and macronutrient intake and GI symptoms in a sample of 120 individuals with ASD, in whom relationships were previously observed and reported between stress reactivity and GI symptoms. These findings suggest that dietary changes do not appear to be driving GI symptoms nor do GI symptoms appear to impact dietary behavior among those with ASD.

## Data Availability

The datasets generated for this study are available on request to the corresponding author.

## Ethics Statement

This study was carried out in accordance with the recommendations of the Institutional Review Boards at the University of Missouri and Vanderbilt University with written informed consent from all participants under the age of 18. All participants gave written informed consent in accordance with the Declaration of Helsinki. The protocol was approved by the Institutional Review Boards at the University of Missouri and Vanderbilt University.

## Author Contributions

BF conceptualized and designed the study, coordinated and supervised data collection, drafted the initial manuscript, and reviewed and revised the manuscript. ShM and DS processed and entered data, conducted a literature review, and assisted with preparation of the manuscript. KD carried out the statistical analyses and assisted with preparation of the manuscript. SaM collected the GI and diet data from patients at Vanderbilt University. JV-V supervised data collection at Vanderbilt University and revised the manuscript. KG, KS, and MB provided their expertise and guidance regarding autism spectrum disorder and revised the manuscript. DB supervised the research team and provided expertise and guidance regarding autism spectrum disorder and gastrointestinal disorders in autism and revised the manuscript. All authors approved of the final manuscript as submitted.

## Funding

This research was supported by a grant given to the Autism Treatment Network, Autism Intervention Research Network on Physical Health by the Health Resources Services Administration (HRSA Grant no. UA3MC11054). This information or content and conclusions are those of the author and should not be construed as the official position or policy of, nor should any endorsements be inferred by HRSA, HHS, or the US Government. This work was conducted through the Autism Speaks Autism Treatment Network serving as the Autism Intervention Research Network on Physical Health.

## Conflict of Interest Statement

The authors declare that the research was conducted in the absence of any commercial or financial relationships that could be construed as a potential conflict of interest.

## Abbreviations

ASD, autism spectrum disorder; GI, gastrointestinal; AS-ATN, Autism Speaks Autism Treatment Network.
